# Naringenin regulates production of matrix metalloproteinases in the knee-joint and primary cultured articular chondrocytes and alleviates pain in rat osteoarthritis model

**DOI:** 10.1590/1414-431X20165714

**Published:** 2017-03-23

**Authors:** C.C. Wang, L. Guo, F.D. Tian, N. An, L. Luo, R.H. Hao, B. Wang, Z.H. Zhou

**Affiliations:** 1 Department II of Orthopedics, Affiliated Zhongshan Hospital of Dalian University, Dalian, Liaoning, China; 2 Department of Respiratory Medicine, Affiliated Zhongshan Hospital of Dalian University, Dalian, Liaoning, China

**Keywords:** Metalloproteinase, Osteoarthritis, Naringenin, Chondrocyte, Knee-joint, Rat MIA model

## Abstract

Inflammation of cartilage is a primary symptom for knee-joint osteoarthritis. Matrix metalloproteinases (MMPs) are known to play an important role in the articular cartilage destruction related to osteoarthritis. Naringenin is a plant-derived flavonoid known for its anti-inflammatory properties. We studied the effect of naringenin on the transcriptional expression, secretion and enzymatic activity of MMP-3 *in vivo* in the murine monosodium iodoacetate (MIA) osteoarthritis model. The assessment of pain behavior was also performed in the MIA rats. The destruction of knee-joint tissues was analyzed microscopically. Moreover, the effect of naringenin was also studied *in vitro* in IL-1β activated articular chondrocytes. The transcriptional expression of MMP-3, MMP-1, MMP-13, thrombospondin motifs (ADAMTS-4) and ADAMTS-5 was also studied in primary cultured chondrocytes of rats. Naringenin caused significant reduction in pain behavior and showed marked improvement in the tissue morphology of MIA rats. Moreover, a significant inhibition of MMP-3 expression in MIA rats was observed upon treatment with naringenin. In the *in vitro* tests, naringenin caused a significant reduction in the transcriptional expression, secretion and enzymatic activity of the studied degradative enzymes. The NF-κB pathway was also found to be inhibited upon treatment with naringenin *in vitro*. Overall, the study suggests that naringenin alleviated pain and regulated the production of matrix-metalloproteinases via regulation of NF-κB pathway. Thus, naringenin could be a potent therapeutic option for the treatment of osteoarthritis.

## Introduction

Millions of people, mostly elderly, are affected by osteoarthritis (OA) all over the world. The symptoms of osteoarthritis include inflammation of synovial joints, osteophyte formation and articular cartilage degeneration. Although, clear understanding of the causes of osteoarthritis remains elusive, several causes such as physiologic synthesis disruption and articular cartilage degradation during osteoarthritis progression have been postulated (1,2).

Matrix metalloproteinases (MMPs) play a central role in articular cartilage destruction in patients with osteoarthritis (3,4). MMPs have been classified into gelatinases (MMP-2, MMP-9), collagenases (MMP-1, MMP-8, MMP-13) and stromelysins (MMP-3, MMP-7, MMP-10, MMP-11) (5,6). Of these MMPs, MMP-3 is most well-known for its role in degradation of proteoglycans and also activation of procollagenase (7,8). Several plant-derived products are known for their anti-arthritic properties (9). Therefore, studies should clinically test any natural remedy that could have an effect on the expression and activity of MMP-3 and other matrix metalloproteinases.

The present therapeutic regimen of osteoarthritis primarily involves pain management by using non-steroidal anti-inflammatory drugs (NSAIDs) (10). Although the NSAID therapy of osteoarthritis is well established, chronic NSAID use may lead to several side effects including gastrointestinal damage (11,12). Therefore, use of natural therapeutic agents along with traditional NSAIDs has been proposed (13).

Several flavonoids are known for their role in alleviation of osteoarthritis (14,15). Naringenin is a plant-derived flavanone found in tomato, grapefruit and citrus fruits (16). Naringenin is known for its antioxidant and anti-inflammatory properties (17,18). Naringenin has also been shown to have a role in modulation of MMP-2 and MMP-9 via inhibition of NF-κB pathway (19). However, the role of naringenin in modulation of MMP-3 in osteoarthritis has not yet been explored. In this study, we explored the chondro-protective activity of naringenin in a rat OA model. Specifically, we identified the effect of naringenin on the alleviation of pain associated with osteoarthritis in monosodium iodoacetate (MIA) murine OA model. The effects of naringenin on *in vivo* MMP-3 secretion and on IL-1β-induced gene expression of several degradative enzymes including MMPs *in vitro* were studied. We also attempted to identify the possible mechanism of regulation of degradative enzyme levels upon induction with IL-1β *in vitro*.

## Material and Methods

Male Wistar rats (220–240 g; n=45) were used to determine the effect of naringenin on the alleviation of OA. The animals were kept under a 12/12 hour light and dark circadian cycle and under controlled conditions of temperature and humidity. The animals were fed standard rat diet and water *ad libitum*. All animals’ up-keeping procedures were carried out according to the guidelines of World Medical Association Animal Ethics Guidelines (declaration of Helsinki).

### Induction of OA in rats and experimental groups

The animals were anesthetized with isoflurane, and then 2 mg MIA (Sigma-Aldrich, USA) was injected in the intra-articular space of the right knee using a 26.5 G needle in 30 µL volume. The control group was injected with phosphate buffered saline (PBS). The animals were assigned to three treatment groups at random with 15 animals per group as follows: 1) control group that was administered with PBS; 2) 20 mg/kg naringenin; and 3) 40 mg/kg naringenin. Naringenin was obtained from Sigma-Aldrich (USA) and dissolved in 5% dimethyl sulfoxide (DMSO) and administered orally by gavage in 300 µL volume doses daily. Naringenin treatment commenced two days after induction of OA by MIA.

### Assessment of pain behavior in MIA rats

A dynamic planter esthesiometer (Ugo Basile, Italy) was used for the nociceptive testing of animals. Testing was performed prior to MIA induction and subsequently each day until the end of the experiment (2 weeks). Before testing, the animals were rested for 30 min in a controlled environment chamber. The touch-stimulator of the instrument was aligned under the animal. The angled mirror was used to orient the stimulating micro-filament below the hind-paw planter surface. Upon activation, the 0.5 mm microfilament progressed to touch the proximal region of the metatarsal area. Starting below detection threshold, a gradually increasing stimulating force was applied by the filament until retraction of the paw occurred indicating pain stimulus. The pain stimulating force was automatically recorded. A maximum force of 500 N and a ramp speed of 20 s were employed in all the tests.

### Effect of naringenin on *in vivo* secretion of MMP-3

The animals were euthanized at the end of treatment using the CO_2_ overdose method. Isolation of the rat knee articular cartilage was performed. For isolation, the tissue was homogenized and protein concentration was estimated using Bradford’s method. Fifteen micrograms of the estimated protein was loaded per well on 12% sodium dodecyl sulfate polyacrylamide gel electrophoresis (SDS-PAGE) gel. The proteins were electrophoretically transferred on a polyvinylidene difluoride (PVDF)-membrane after completion of the gel run. Blocking of the membranes was performed for 1.5 h in Tris-buffered saline, which contained 0.2% Tween 20 (TBST) and 2% non-fat dry milk. MMP-3 antibodies were obtained from Cell Signaling Technology, Inc. (USA). The binding of antibodies onto membranes was performed by incubating it with primary antibody solution in TBST and 2% non-fat dry milk for 8 h at 4°C. The membrane was washed thrice with TBST. Finally, the membranes incubated for 1 h with horseradish peroxidase (HRP) conjugated secondary antibodies and developed using an ECL detection system. The band density on the membranes was scanned and then analyzed using the ImageJ (NIH, USA) software program.

### Histopathological analysis of rat knee

The knee tissues were isolated from sacrificed animals and fixed in 12% neutral buffered formaldehyde solution for 72 h at 4°C. The decalcification of tissues was performed in formic acid for 6 days. Subsequently, the tissue was dehydrated in increasing concentration of ethanol. The dehydrated tissues were cleared with xylene and fixed in paraffin. Sections of five to seven microns were cut using microtome and stained with hematoxylin and eosin. The morphological differences between the sections of different treatment groups were scored following the modified Mankin scoring system. For scoring, morphological characteristics were analyzed as follows: N: normal morphology, +: mild damage, ++: severe damage. The scoring was performed twice by two independent researchers who were blind to the treatment experiment.

### Primary culture of articular chondrocytes and treatment with naringenin

Articular tissues of the animals were isolated from femoral condyle and tibial plateau. The isolated tissues were washed with PBS, ground to small pieces and digested with 0.3 % solution of collagenase for 3 h at 37°C. The digested homogenate was centrifuged at 10,000 *g* for 1 min at 4°C and the cell pellet was collected. The individual cells were transplanted to 100 mm culture plates with a seeding density of 10^5^ cells/cm^2^ in 10 mL of DMEM, 12 % fetal bovine serum, penicillin (100 units/mL) and streptomycin (100 μg/mL). The cells were cultured under 5 % CO_2_/95 % air at 37°C. The culture medium was replaced daily.

The chondrocytes were seeded on 6-well culture plates at a culture density of 10^5^ cells/cm^2^. After 2 days of culture, the cells were incubated with 20 and 40 μM of naringenin for 3 h. Subsequently, the cells were incubated in IL-1β (20 ng/mL) for 24 h with a negative control of absence of IL-1β. Naringenin solution was prepared in dimethyl sulfoxide (DMSO) and PBS. The final concentration of DMSO was kept at 0.75% and pH 7.0–7.5. No effect of culture medium was found on the various parameters of MMP-3 activity.

### Isolation of total RNA and qRT-PCR

Total RNA was isolated from chondrocytes using the RNeasy Mini Kit (Qiagen, USA). Subsequently, qRT-PCR was performed using One-Step qRT-PCR Kit (Qiagen) following the manufacture’s protocol. The designing of qRT-PCR primers was done using Primer-BLAST (21). The melting temperature (Tm) of primers was taken between 57°C and 63°C. The size of amplification product was taken between 90 and 120 bp along with difference in Tm of 3°C (max). We took three technical replicates for every biological replicate. The quality of RNA was assessed by a QIAxpert™ microfluidic UV/VIS spectrophotometer (Qiagen). The PCR master mix included 5 µL DyNAmo Flash SYBR Green (Thermo) (2X), 1.5 µL cDNA, 1 µL (5 pm/µL) each primer. The following cycling conditions were used for qRT-PCR: denaturation at 95°C for 10 min, 40 denaturation cycles at 95°C for 22 s, annealing with extension at 60°C for 60 s. The qRT-PCR amplification was performed in ABI 7500 system (Applied Biosystems, USA). The threshold cycle value (Ct) for the genes were quantified and normalized by Ct value for glyceraldehyde-3-phosphate dehydrogenase (GADPH) expression. The following primers were used for qRT-PCR: MMP-3 (5′-TTTGGCCGTCTCTTCCATCC-3′, 5′-GGAGGCCCAGAGTGTGAATG-3′), MMP-13 (5′-GGACTCACTGTTGGTCCCTG-3′; 5′-GGATTCCCGCAAGAGTCACA-3′), MMP-1 (5′-CCGGCAGAATGTGGAAACAG-3′, 5′-GCTGCATTTGCCTCAGCTTT-3′), ADAMTS-4 (5′-CATCCTACGCCGGAAGAGTC-3′, 5′-AAGCGAAGCGCTTGTTTCTG-3′), and ADAMTS-5 (5′-CCCAAATACGCAGGTGTCCT-3′; 5′-ACACACGGAGTTGCTGTAGG-3′). GAPDH (5′-TGTGAACGGATTTGGCCGTA-3′; 5′-TGAACTTGCCGTGGGTAGAG- 3′) was used as an internal control.

### Assay for assessment of cytotoxicity of naringenin

The chondrocytes were seeded on a 96-well microtiter plate at a density of 2×10^5^ cells/mL and were allowed to attach onto the surface for 24 h, which allowed optimal treatment with naringenin during the log-phase of cell growth. Naringenin solution was prepared in DMSO in a graded set of solutions ranging from 10 to 100 µM. Treatment with naringenin was performed in DMEM and 10% FBS. The cells were incubated for 72 h and the proliferation of cells was estimated by the sulforhodamine-B (SRB) assay (20).

### Estimation of MMP-3 and phosphorylated forms of NF-κB and IκB-α levels in the articular chondrocytes culture supernatants

Antibodies for NF-κB p65 and p-NF-κB p65 were obtained from Santa Cruz Biotechnology, Inc. (USA) and anti-MMP-3, anti-IκB-α and anti-p-IκB-α antibodies were obtained from Cell Signaling Technology, Inc. The concentrations of the proteins in the culture supernatants were estimated using Bradford’s method as mentioned above. Fifteen micrograms of the estimated protein was loaded per well on 12% SDS-PAGE gel. Western blot densitometry was performed as described above.

### Estimation of MMP-3 proteolytic activity

Chondrocyte culture supernatants were pretreated for 3 h with naringenin. Subsequently, IL-1β (20 ng/mL) was used for stimulation of these culture supernatants in medium containing DMEM and FBS (0.5%). Protein concentrations were estimated using Bradford’s method and 15 μg of the estimated protein was loaded per well on 12% SDS-PAGE gel with casein (0.1%), 10 mM Tris-HCl at pH 8.0. The gels were washed with 10 mM Tris-HCl (pH 8.0) containing 2% Triton X-100 after electrophoresis. After washing, the gels were incubated in 100 mM Tris-HCl, pH 8.0, with 2% Triton-X100, 0.1 M NaCl, 4 mM CaCl_2_ for 48 h at 37°C. Coomassie blue staining of the gels was performed and the band density on the membranes was scanned and then analyzed using the ImageJ (NIH, USA) software program.

### Statistical analysis

The mean values of the individual experimental groups were calculated as percentage of the control group. The individual averages are reported as average±SEM. The data for assessment of pain behavior are reported as average±SEM. The variance between the experimental groups was estimated using one-way ANOVA with Bonferonni’s multiple corrections for *post hoc* analysis. P≤0.05 was considered to be significant. All statistical analyses were performed in Statistica v8.0 (StatSoft, USA).

## Results

### Effect of naringenin treatment on pain generation in OA rats

**Figure 1:**
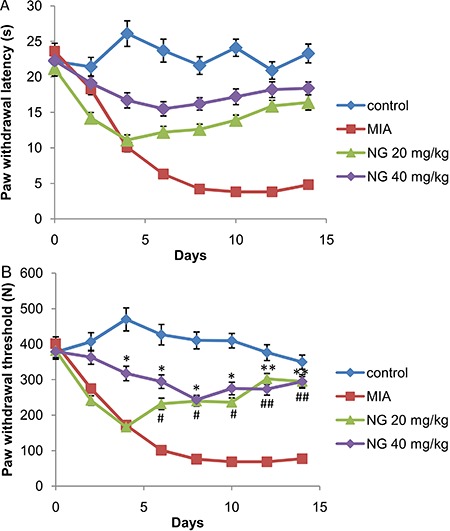
Effect of naringenin (NG) on mechanical hyperalgesia in a model of monosodium iodoacetate (MIA)-induced osteoarthritis in rats. Two milligrams of MIA was injected, and 2 days later, NG was administered orally for 2 weeks at 20 and 40 mg/kg. *A*, Paw withdrawal latency (s). *B*, Paw withdrawal threshold (N). Data are reported as means±SEM. *P≤0.05 and **P≤0.01 compared to MIA group; ^#^P≤0.05 and ^# #^P≤0.01 compared to MIA group (ANOVA with Bonferonni’s *post hoc* multiple corrections).

In the pain assessment test, parameters such as paw withdrawal latency (PWL) and paw withdrawal threshold (PWT) showed a significant prolongment upon treatment with naringenin when compared with the MIA group (Figure 1). These results showed that pain alleviation began from the fourth day of the treatment. It was also found that at a dose of 40 mg/kg the effect of naringenin was more pronounced and significant compared to the 20 mg/kg naringenin group.

### Effect of naringenin on *in vivo* secretion of MMP-3

**Figure 2:**
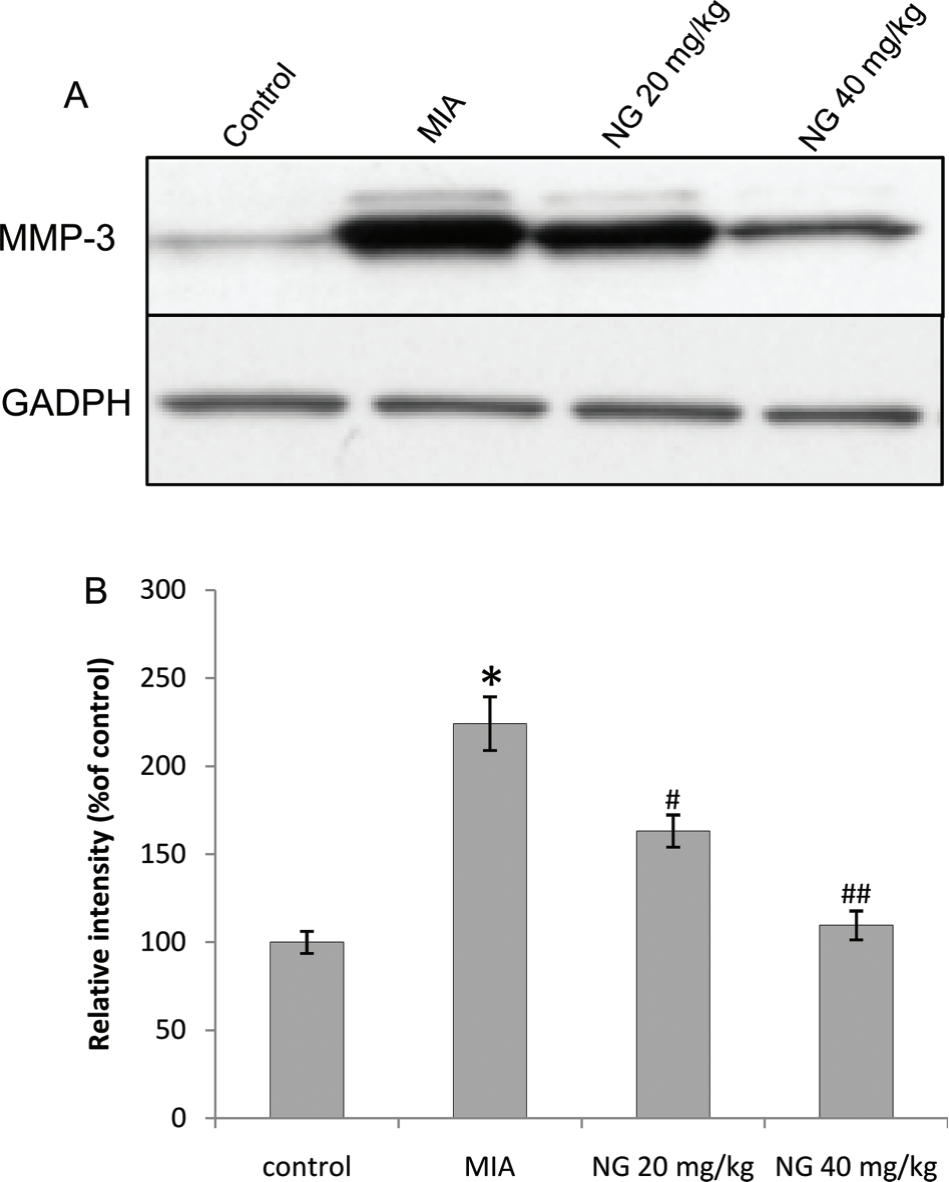
Effect of naringenin (NG) on the production of MMP-3 *in vivo*. Naringenin was administered orally for 2 weeks at 20 and 40 mg/kg, beginning 2 days post-induction of osteoarthritis by 2 mg of monosodium iodoacetate (MIA). Total protein was isolated from rat knee articular cartilage. MMP-3 concentration was estimated using western blot densitometry. *A*, Western blot of MMP-3 and GADPH; *B*, relative intensity of bands. Data are reported as means±SEM. *P≤0.05, compared to negative control (PBS); ^#^P≤0.05 and ^# #^P≤0.01, compared to MIA-treated group (ANOVA with Bonferonni’s *post hoc* multiple corrections).

To determine the effect of naringenin on *in vivo* expression of MMP-3 in the articular cartilage tissues, western blot densitometry for MMP-3 was performed. In the MIA rats, there was a significantly greater MMP-3 activity compared to control animals (Figure 2). Upon treatment with naringenin, a significant decrease in MMP-3 activity was observed. The greatest decrease was observed at 40 mg/kg naringenin.

### Effect of naringenin on knee histopathology

**Figure 3:**
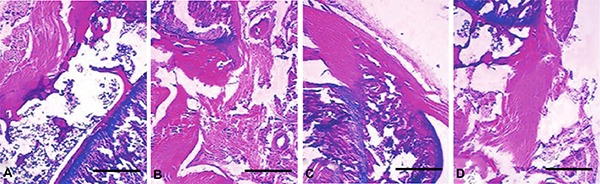
Effect of naringenin on histopathology of knee tissue of monosodium iodoacetate (MIA) rats. Naringenin was administered orally for 2 weeks at 20 and 40 mg/kg beginning 2 days post-induction of osteoarthritis by 2 mg of MIA. The knee tissue was collected at the end of treatment (2 weeks). *A*, Negative control, *B*, MIA-treated, *C*, naringenin 20 mg/kg, *D*, naringenin 40 mg/kg. See Table 1 for the results of Mankin scoring analysis. Magnification bar: 200 µM.

The effect of oral administration of naringenin on MIA induced-osteoarthritis is shown in Table 1. The induction of OA caused mild to severe chondrocyte death, degeneration of muscle tissue, erosion of cartilage and fibrillation (Figure 3) compared to the control group. The administration of 20 mg/kg naringenin caused sub-maximal effect on those parameters. However, administration of 40 mg/kg naringenin showed marked improvement in the knee tissue morphology. A significant attenuation of chondrocyte death, muscle degradation, cartilage erosion and fibrillation was observed in knee tissues at 40 mg/kg.

**Table 1:**
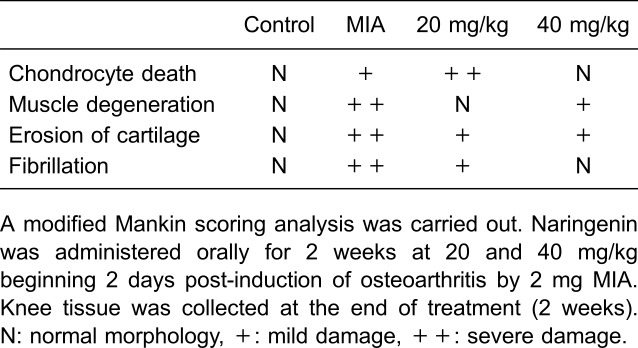
Effect of naringenin on morphology of knee tissue of murine monosodium iodoacetate-induced (MIA) osteoarthritis in rats.

### Effect of naringenin on the transcriptional expression of MMP-3, MMP-1, MMP-13, ADAMTS-4, and ADAMTS-5

**Figure 4:**
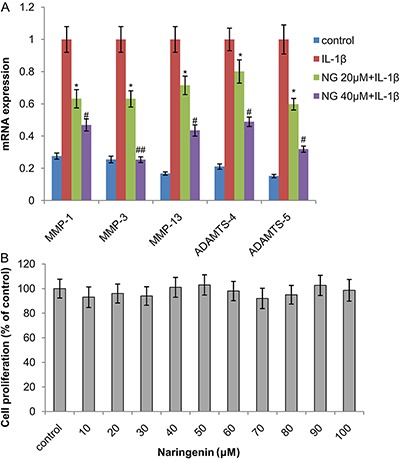
*A*, Effect of naringenin (NG) on gene expression of MMP-1, MMP-3, MMP-13, ADAMTS-4, and ADAMTS-5 in rat chondrocytes. The rat articular chondrocytes were cultured for 2 days and were incubated with 20 and 40 μM of NG for 3 h. Subsequently, the cells were incubated in IL-1β (20 ng/mL) for 24 h with a negative control of absence of IL-1β. *B*, Effect of NG on proliferation of rat chondrocytes. Seeding of chondrocytes was performed (2×10^5^ cells/mL) for 24 h under varying concentrations of naringenin ranging from 10 to 100 μM. The proliferation of cells was estimated by sulforhodamine-B (SRB) assay. Data are reported as means±SEM. *P≤0.05, compared to IL-1 β; ^#^P≤0.05 and ^# #^P≤0.01, compared to IL-1β (ANOVA with Bonferonni’s *post hoc* multiple corrections).

We determined the effect of naringenin on the expression of MMPs and thrombospondin motifs in IL-1β stimulated chondrocyte cells. The expression of MMP-3, MMP-1 and MMP-13 showed a significant reduction in naringenin-treated cells. Similarly, the expression of ADAMTS-4, and ADAMTS-5 was also significantly reduced upon treatment with naringenin of IL-1β-treated chondrocytes. In all cases, gene expression of MMP-1, MMP-3, MMP-13, ADAMTS-4 and ADAMTS-5 showed a significant decrease with naringenin treatment (Figure 4A). In this analysis, the effect of naringenin was also found to be dose-dependent.

### Effect of naringenin on chondrocyte proliferation

The cytotoxicity of naringenin on rat articular chondrocytes was estimated from 10 to 100 µM concentration. No significant difference in cytotoxicity was found for naringenin compared to control (Figure 4B). Moreover, at all concentrations, the numbers for cell proliferation was almost the same without a dose-dependent trend.

### Effect of naringenin on IL-1β-induced secretion of MMP-3 from rat articular chondrocytes

**Figure 5:**
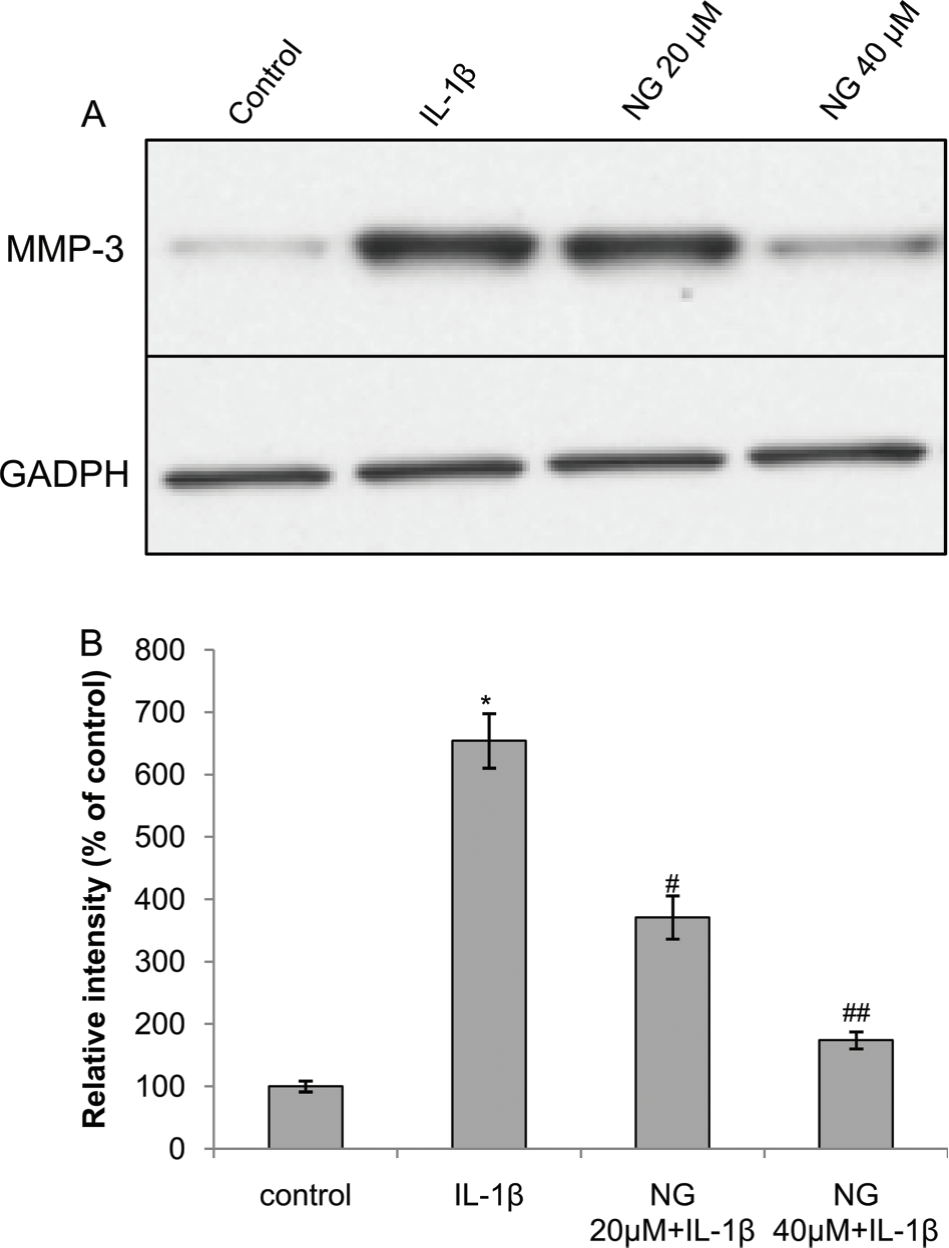
Effect of naringenin on MMP-3 secretion in rat chondrocytes. Primary cultured articular chondrocytes were pretreated with 20 and 40 μM of naringenin. MMP-3 concentration was estimated in the total protein of articular chondrocytes using western blot densitometry. *A*, Western blot of MMP-3 and GADPH; (*B*) relative intensity of bands. Data are reported as means±SEM. *P≤0.05, compared to negative control (PBS); ^#^P≤0.05 and ^#^P≤0.01, compared IL-1β-treated culture (ANOVA with Bonferonni’s *post hoc* multiple corrections).

A significant decrease in gene expression of MMP-3 upon treatment with naringenin was found. Therefore, western blot analysis was performed to identify if this transcriptional change was also observable in translation. There was a large increase in MMP-3 production upon IL-1β induction (Figure 5). Treatment with naringenin caused a significant decrease in the protein concentration of MMP-3 in a dose-dependent manner.

### Effect of naringenin on phosphorylated forms of NF-κB and IκB-α

**Figure 6:**
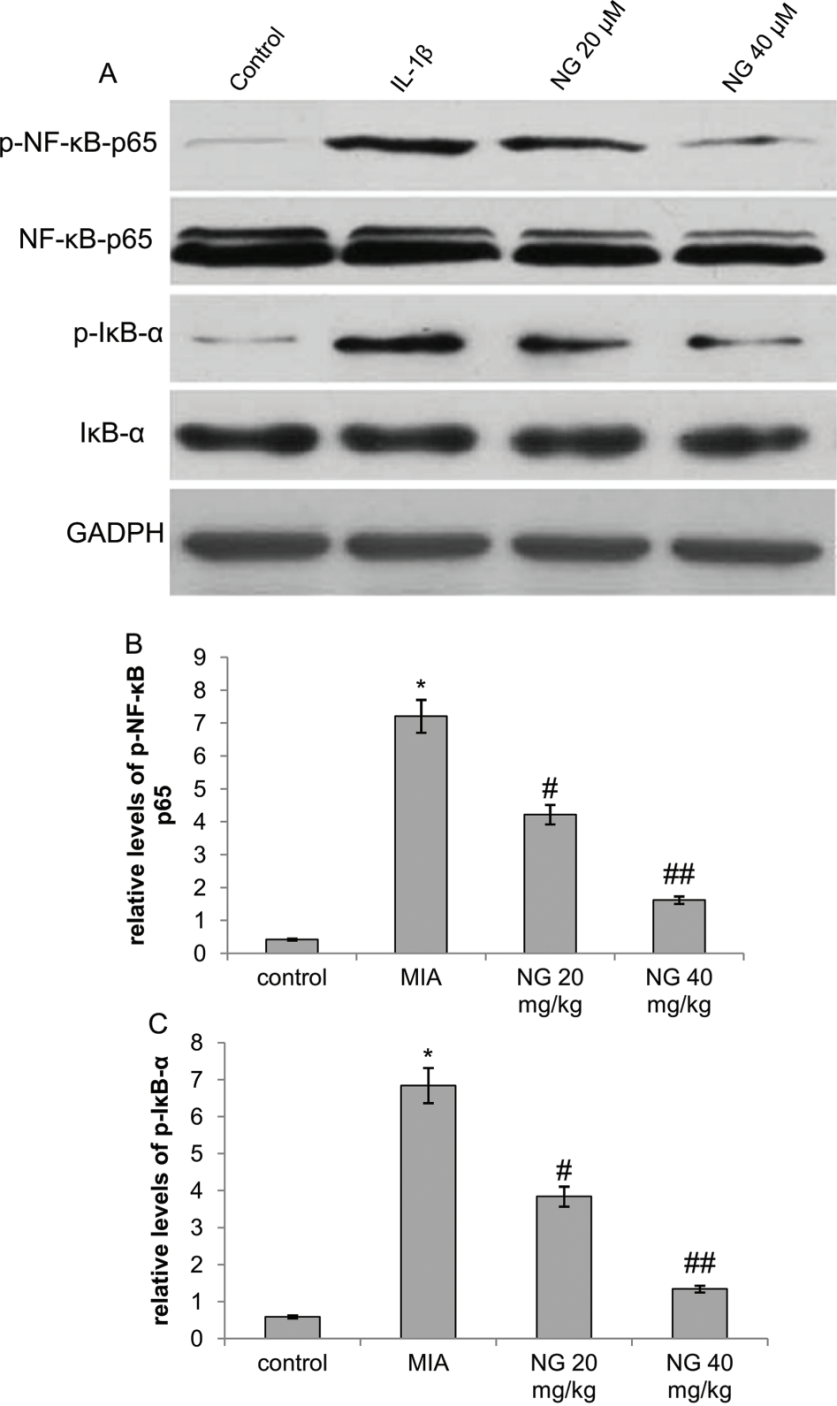
Effect of naringenin phosphorylated forms of NF-κB and IκB-α. Primary cultured articular chondrocytes were pretreated with 20 and 40 μM of naringenin. *A*, Western blot of NF-κB p65, p-NF-κB p65, IκB-α and p-IκB-α and GADPH (control); (*B*) relative intensity of bands of p-NF-κB p65; (*C*) relative intensity of bands of p-IκB-α. MIA: monosodium iodoacetate. Data are reported as means±SEM. *P≤0.05, compared to negative control (PBS); ^#^P≤0.05 and ^# #^P≤0.01, compared IL-1β treated culture (ANOVA with Bonferonni’s *post hoc* multiple corrections).

The *in vivo* protein expression of NF-κB and IκB-α and their phosphorylated forms was estimated using western blot densitometry. As illustrated in Figure 6, OA induction with MIA resulted in 17- and 11-fold increases (P<0.01) in the phosphorylation of NF-κB p65 and IκB-α, respectively, compared to the control group. However, a significant and dose-dependent decrease in the NF-κB p65 and IκB-α phosphorylation was observed upon treatment with naringenin. These results indicate that naringenin could inhibit the NF-κB activation due to OA induction.

### Effect of naringenin on proteolytic activity of MMP-3 in rat articular chondrocytes

**Figure 7:**
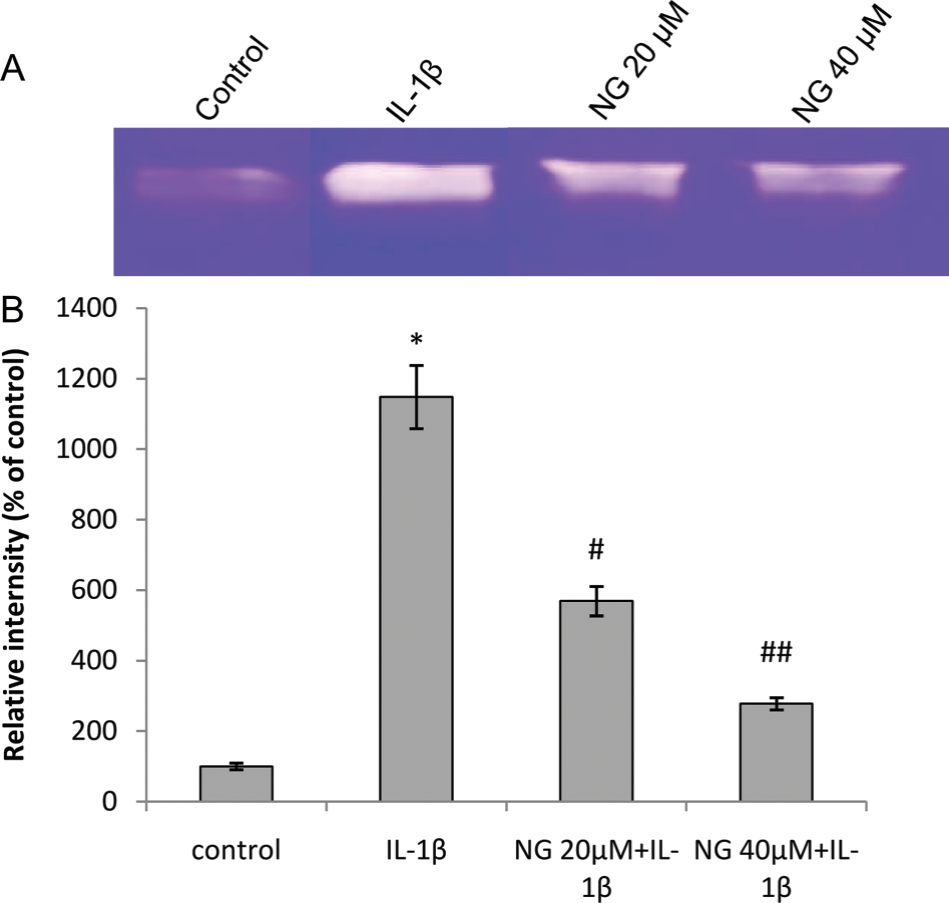
Effect of naringenin on caseinolytic activity of MMP-3 secretion in rat chondrocytes. Primary cultured articular chondrocytes were pretreated with 20 and 40 μM of naringenin. MMP-3 activity was estimated with casein zymography (*A*) Coomassie blue-stained polyacrylamide gel of casein zymography; (*B*) relative intensity of bands. Data are reported as means±SEM *P≤0.05, compared to negative control (PBS); ^#^P≤0.05 and ^# #^P≤0.01, compared IL-1β treated culture (ANOVA with Bonferonni’s *post hoc* multiple corrections).

To determine the effect of naringenin on the enzymatic activity of MMP-3, we determined the caseinolytic activity of MMP-3 in the rat articular chondrocytes using casein zymography. We found a large and significant increase in the MMP-3 activity upon IL-1β induction (Figure 7). This induction showed a significant decrease upon pre-treatment with naringenin in a dose-dependent manner as was evident from the Coomassie blue stained SDS-PAGE gel.

## Discussion

Osteoarthritis is a degenerative disease of articular chondrocytes and involves several inflammatory pathways and mediators such as IL-6, IL-1β and NO (22). MMPs are known to degrade the extracellular matrix components and elevated activity of MMPs has been associated with degradation of cartilage in OA patients. IL-1β is a proinflammatory cytokine, which causes up-regulation of MMPs in the articular chondrocytes, contributing to destruction of cartilages (23-25). Naringenin is a plant-derived flavonoid known to possess anti-inflammatory properties. However, no study has been performed on the identification of its effect on alleviation of OA. In this study, for the first time, we report the effect of naringenin on the expression, production and protein degradation activity of MMPs *in vivo* in MIA rat model as well as *in vitro* in IL-1β-induced MMP production in cultured chondrocytes. We also report the alleviation of pain after treatment with naringenin in MIA rats.

The patho-physiology of pain related to OA is poorly understood. Cartilage is devoid of nerve endings and, thus, it is not the source of pain. OA-related pain arises from afferent nerve activation in the synovium, joint capsule, ligaments, periosteum etc. (26). Pain alleviation is an important goal in the treatment of OA. MIA-induced OA model is well characterized for joint pain (27), and mechanical hyperalgesia is exhibited by this model. The development of lesions in subchondral bone is similar to such lesions and pain in human osteoarthritis (28–30). Thus, it can be said that the attenuation of OA symptoms in the knee-joint tissues observed in the histopathological analysis was in accordance with the pain-reducing effect of naringenin.

In the *in vivo* experiments, naringenin was found to significantly reduce the MMP-3 secretion in the articular cartilage. These results indicated that naringenin treatment can alleviate the OA symptoms by inhibiting the MMP-3 mediated articular cartilage degeneration when administered orally. In addition, the *in vivo* MMP-3 levels were also significantly reduced in the cell cultures of articular chondrocytes induced by IL-1β, as determined by the gene expression levels and western blot densitometry. Moreover, MMP-1 and MMP-13 also showed significant transcriptional down-regulation when treated with naringenin. MMP-1 is generally found in the synovial fluid of OA patients (31–33). In addition to MMPs, the degradative enzymes ADAMTS-4 and ADAMTS-5 also showed significant down-regulation. ADMTS-4 and ADMTS-5 are known to cause destruction of matrix in OA (34,35).

MMP-3 is also known to degrade proteoglycans (8). In order to estimate the effect of naringenin pre-treatment of the Il-1β-induced articular chondrocytes, casein zymography test was performed. A marked decrease in the proteolytic activity of MMP-3 was observed with naringenin pre-treatment. These results indicated that, in addition to reduce expression and secretion of MMP-3, naringenin also reduced the proteolytic potential of MMP-3.

NF-κB is present in the cytoplasm in association with IκB. Abundance of the phosphorylated form of NF-κB and IκB has been reported in OA patients (36). Several studies have shown that NF-κB mediates the expression of MMP-1, MMP-3 and MMP-13 induced by cytokines such as IL-1β (37). In this study, we found an elevated expression of the phosphorylated form of NF-κB and IκB *in vitro* in culture supernatants after induction with IL-1β. This expression was significantly reduced upon treatment with naringenin. Several studies have reported the inhibition of NF-κB pathway by flavonoids including naringenin (38,39). Therefore, our results indicate an involvement of NF-κB pathway in the up-regulation of MMPs upon induction of OA. Furthermore, naringenin may have caused inhibition of the NF-κB pathway causing down-regulation of MMPs.

The present study showed the chondro-protective effect of naringenin on the expression and secretion of several degradative enzymes, particularly MMP-3. The enzymatic activity of MMP-3 was also found to be markedly decreased due to naringenin treatment, both *in vivo* and *in vitro*. However, naringenin showed no significant cytotoxicity on articular chondrocytes culture *in vitro*. Our results also indicate that the down-regulation of MMPs with naringenin treatment was putatively mediated by the NF-κB pathway. However, further studies are required to identify the mechanism by which naringenin causes inhibition of NF-κB pathway. In conclusion, naringenin can be a potential natural therapeutic agent for the treatment of osteoarthritis. Therefore, clinical studies on the effect of naringenin on human osteoarthritis should be planned.
